# A Case of Loeys-Dietz Syndrome Treated With Emergency Endovascular Repair for a Ruptured Internal Iliac Artery Aneurysm

**DOI:** 10.7759/cureus.114443

**Published:** 2026-08-12

**Authors:** Sanayuki Matsuoka, Ryota Asano, Makiko Masuda, Mamina Miyabayashi, Susumu Fujii

**Affiliations:** 1 Cardiovascular Surgery, Ogikubo Hospital, Suginami, JPN; 2 Radiology, Ogikubo Hospital, Suginami, JPN; 3 Radiology, Tokyo Saiseikai Central Hospital, Minato, JPN

**Keywords:** coil embolization, endovascular repair, internal iliac artery aneurysm, loeys-dietz syndrome, n-butyl-2-cyanoacrylate (nbca)

## Abstract

We report the case of a 38-year-old man with Loeys-Dietz syndrome (LDS) who presented in hemorrhagic shock due to rupture of a right internal iliac artery aneurysm, with contrast-enhanced computed tomography (CT) also revealing multiple aneurysms involving the aortic root, abdominal aorta, and several visceral and pelvic arteries. Because of hemodynamic instability and a history of prior abdominal surgery, open surgical repair was considered high-risk, and emergency endovascular treatment was chosen instead. Coil embolization was performed at the proximal neck of the internal iliac artery, and the distal branch vessel was embolized with a mixture of n-butyl-2-cyanoacrylate (NBCA) and Lipiodol, achieving complete hemostasis without unintended embolization. The patient's hemodynamic status stabilized, and he was discharged ambulatory on postprocedure day 30. Genetic testing subsequently confirmed a missense mutation in transforming growth factor-beta receptor type 1 (TGFBR1), establishing the diagnosis of LDS. This case illustrates that combined NBCA and coil embolization can be an effective and rapid hemostatic strategy for a ruptured internal iliac artery aneurysm in patients with LDS, for whom open repair carries substantial risk.

## Introduction

Loeys-Dietz syndrome (LDS) is a hereditary vascular disorder caused by dysregulated transforming growth factor-beta (TGF-β) signaling [1], potentially leading to aneurysm formation not only in the aorta but also in peripheral arteries. Because of the fragility of the vessel wall, rupture can occur even in aneurysms of small diameter [2]. However, the long-term outcomes of endovascular treatment in patients with LDS remain unclear, and careful judgment is required when determining treatment indications. Here, we report a case of coil embolization performed for a ruptured internal iliac artery aneurysm.

## Case presentation

A 38-year-old man presented with right lower extremity pain. His medical history included hypertension diagnosed at a young age and distal pancreatectomy with splenectomy for a pancreatic cyst at age 19. He had a smoking history. His family history was notable for the death of his mother and older brother due to vascular disease.

**Figure 1:**
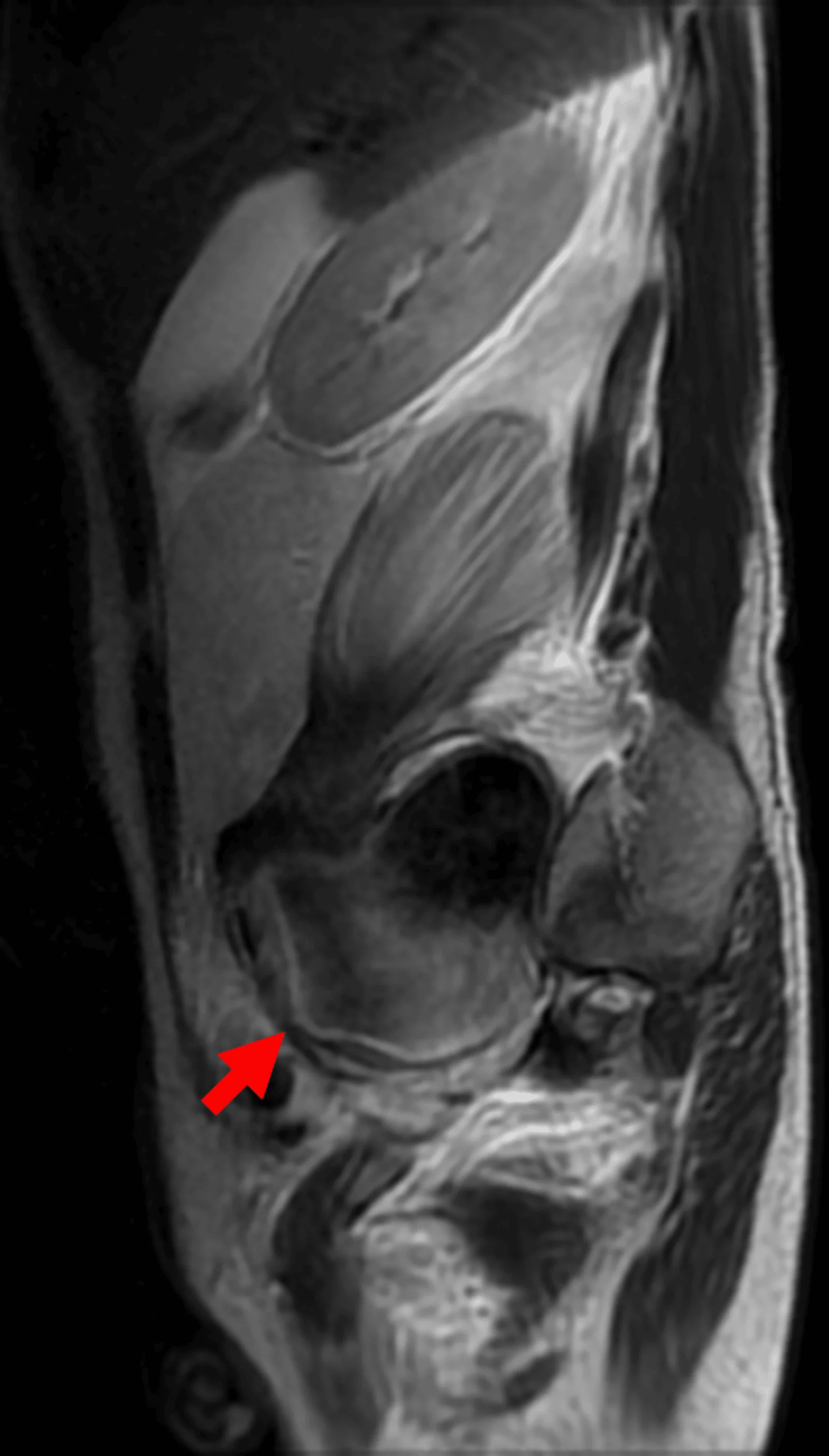
MRI at the referring clinic MRI: magnetic resonance imaging Sagittal MRI obtained during initial evaluation. The red arrow indicates the ruptured internal iliac artery aneurysm

Three days before admission, the patient developed right lower extremity pain, muscle weakness, and sensory dysesthesia, for which he was treated at a local orthopedic clinic. As his symptoms progressively worsened, magnetic resonance imaging (MRI) was performed for further evaluation, during which he developed shock (Figure 1). Noncontrast computed tomography (CT) revealed rupture of the right internal iliac artery aneurysm, and he was transferred to our hospital by emergency ambulance.

On admission, his height was 176 cm, and his weight was 68 kg. His vital signs were as follows: blood pressure, 67/42 mmHg; heart rate, 137 beats per minute; respiratory rate, 46 breaths per minute; oxygen saturation, 100% on room air; and body temperature, 35.8°C (96.4°F). He appeared pale with decreased consciousness and cold extremities. No cardiac murmur or abnormal lung sounds were noted. Facial examination revealed hypertelorism, and physical examination showed arachnodactyly and joint hypermobility.

**Figure 2:**
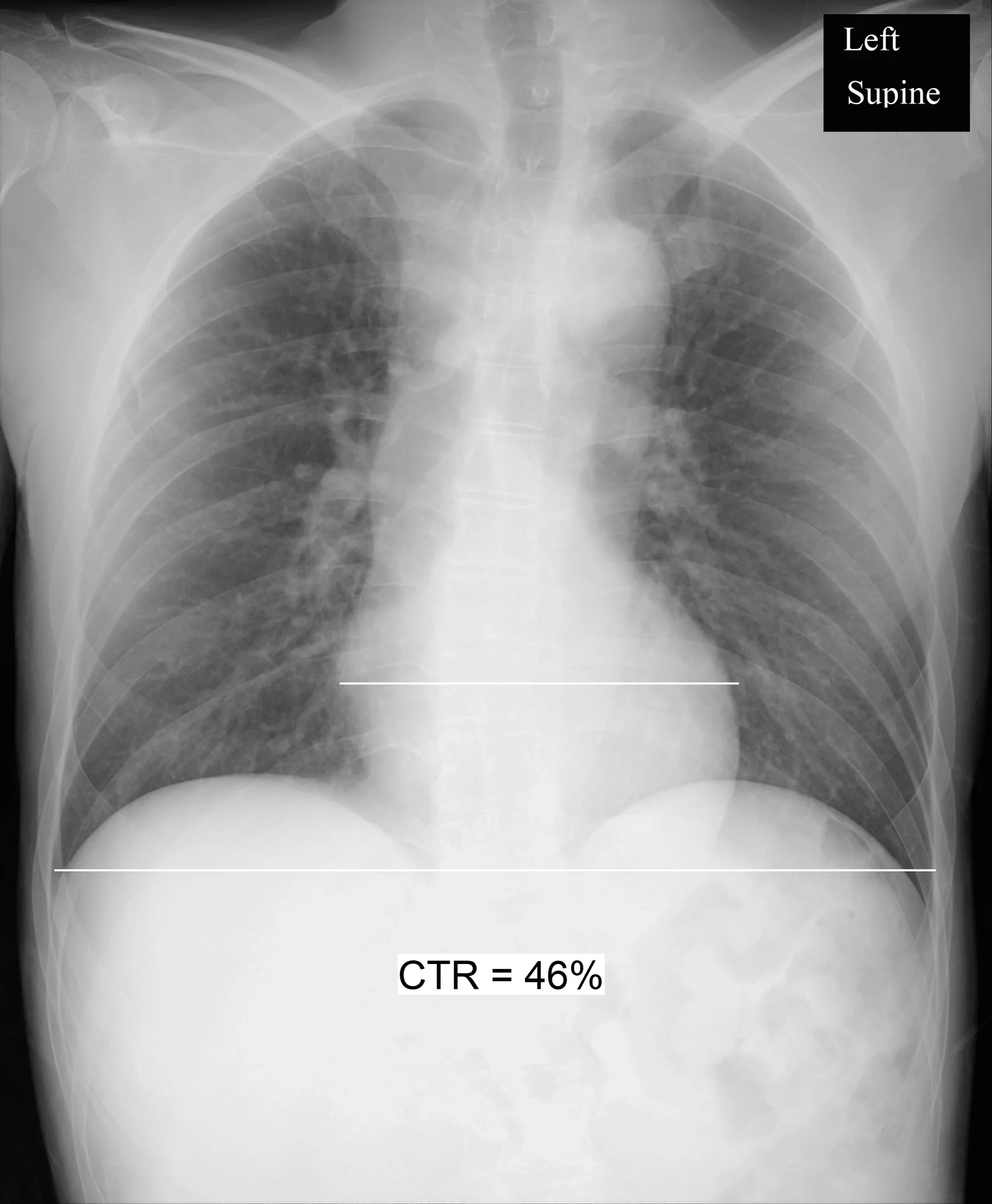
Chest radiograph on admission CTR: cardiothoracic ratio Chest radiography showed a CTR of 46% without pulmonary congestion, along with widening of the mediastinum

**Figure 3:**
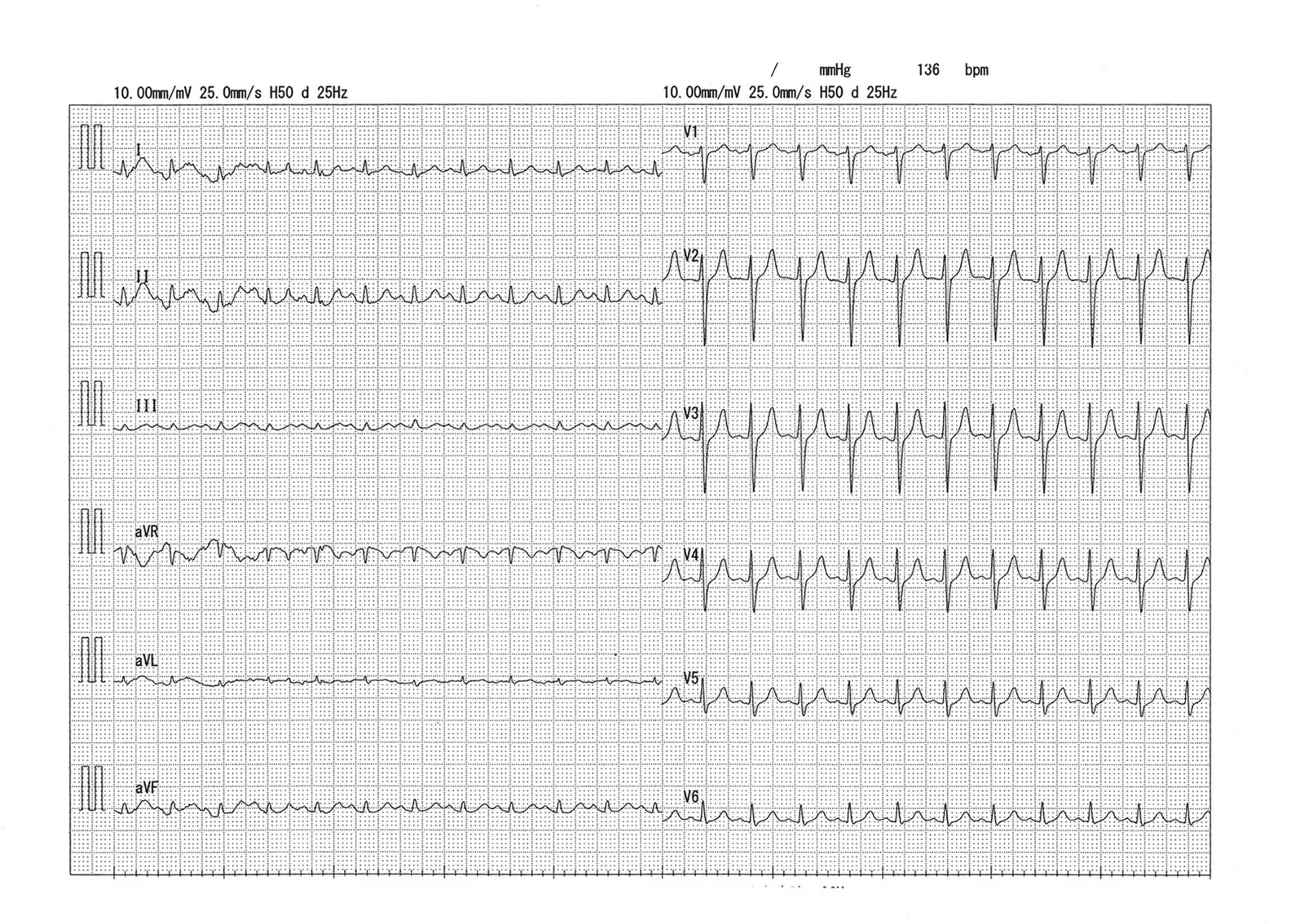
Electrocardiogram on admission Electrocardiography showed sinus rhythm with a heart rate of 136 bpm and no ST-segment changes

Laboratory findings on admission are summarized in Table 1. Chest radiography showed a cardiothoracic ratio of 46% without pulmonary congestion, along with widening of the mediastinum (Figure 2), and electrocardiography showed sinus rhythm with a heart rate of 136 bpm and no ST-segment changes (Figure 3).

**Table 1:** Laboratory findings on admission WBC: white blood cell count; Hb: hemoglobin; Ht: hematocrit; Plt: platelet count; FDP: fibrin degradation products; AST: aspartate aminotransferase; ALT: alanine aminotransferase; LDH: lactate dehydrogenase; γ-GTP: gamma-glutamyl transpeptidase; CK: creatine kinase; BUN: blood urea nitrogen; Cre: creatinine; Glu: glucose; AMY: amylase; Alb: albumin; Na: sodium; K: potassium; CRP: C-reactive protein Reference ranges are standard adult intervals and may vary by laboratory

Parameter	Result	Reference range
WBC	23,000/μL	3,300-8,600/μL
Hb	8.2 g/dL	13.7-16.8 g/dL
Ht	38.6 %	40.7-50.1 %
Plt	300,000 /μL	158,000-348,000 /μL
FDP	21.9 μg/mL	<5 μg/mL
D-dimer	10.2 μg/mL	<1.0 μg/mL
AST	15 U/L	13-30 U/L
ALT	14 U/L	10-42 U/L
LDH	145 U/L	124-222 U/L
γ-GTP	36 U/L	13-64 U/L
CK	119 U/L	59-248 U/L
Total bilirubin	0.8 mg/dL	0.4-1.5 mg/dL
BUN	14.5 mg/dL	8-20 mg/dL
Cre	1.15 mg/dL	0.65-1.07 mg/dL
Uric acid	4.4 mg/dL	3.7-7.8 mg/dL
Glu	303 mg/dL	73-109 mg/dL
AMY	34 U/L	44-132 U/L
Alb	2.7 g/dL	3.9-4.9 g/dL
Na	138 mmol/L	138-145 mmol/L
K	3.9 mmol/L	3.6-4.8 mmol/L
Lactate	5.9 mmol/L	0.5-1.6 mmol/L
CRP	<0.05 mg/dL	<0.14 mg/dL

**Figure 4:**
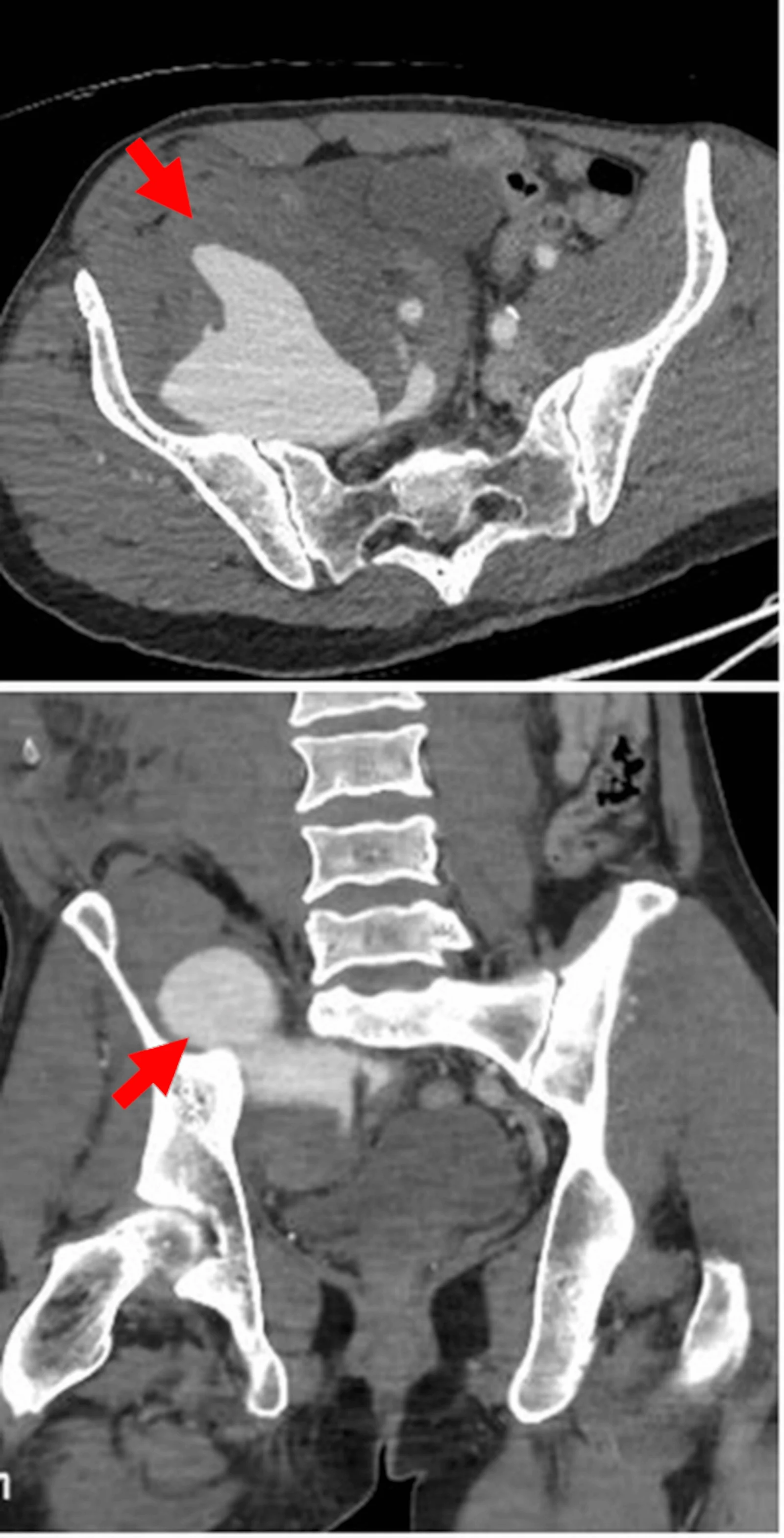
Ruptured right internal iliac artery aneurysm CT: computed tomography Contrast-enhanced CT (axial and coronal views) showing an 88 mm ruptured aneurysm arising from the right internal iliac artery, with an extensive right retroperitoneal hematoma indicating active rupture. The red arrow indicates the ruptured internal iliac artery aneurysm

Contrast-enhanced CT demonstrated rupture of the right internal iliac artery with formation of an 88 mm ruptured aneurysm and a retroperitoneal hematoma extending into the right retroperitoneal space (Figure 4). Diffuse irregular narrowing and dilatation of the systemic arteries were also observed, along with multiple additional aneurysms, including an aortic root aneurysm (48 mm), an abdominal aortic aneurysm (32 mm), bilateral common iliac artery dilatation (maximum diameter 24 mm on the right, 22 mm on the left), multiple bilateral internal thoracic artery aneurysms (maximum diameter 7 mm), a hepatic artery aneurysm (maximum diameter 6 mm), multiple superior mesenteric artery aneurysms (maximum diameter 10 mm), an inferior mesenteric artery aneurysm (maximum diameter 3 mm), a fusiform aneurysm of the right superior gluteal artery (maximum diameter 8 mm), a left internal iliac artery aneurysm (maximum diameter 11 mm), and multiple aneurysms of the right inferior gluteal artery (maximum diameter 7 mm).

Based on these findings, the patient was diagnosed with rupture of a right internal iliac artery aneurysm secondary to an underlying hereditary vascular disorder. Given his hemodynamic instability and history of prior abdominal surgery, open surgical repair was considered high-risk, and endovascular treatment was selected instead.

Procedure

Under local anesthesia with 1% lidocaine (Xylocaine), the left femoral artery was punctured, and a sheath was inserted. The right internal iliac artery was accessed, and a microcatheter was advanced distal to the site of rupture. A mixture of n-butyl-2-cyanoacrylate (NBCA) and Lipiodol (NBCA:Lipiodol ratio of 1:2), totaling 1.5 mL, was rapidly injected into the aneurysm to embolize the distal branch artery (superior gluteal artery). Subsequently, a total of eight coils were deployed from the proximal side of the rupture site to the ostium of the right internal iliac artery: one Ruby coil (8 mm × 35 cm), two Ruby coils (16 mm × 35 cm and 16 mm × 60 cm), two Penumbra occlusion device (POD) packing coils (30 cm each), two Target XL coils (5 mm × 15 cm each), and one Ruby coil (4 mm × 15 cm). Final angiography confirmed absence of blood flow within the aneurysm, and the procedure was completed.

Postoperative course

**Figure 5:**
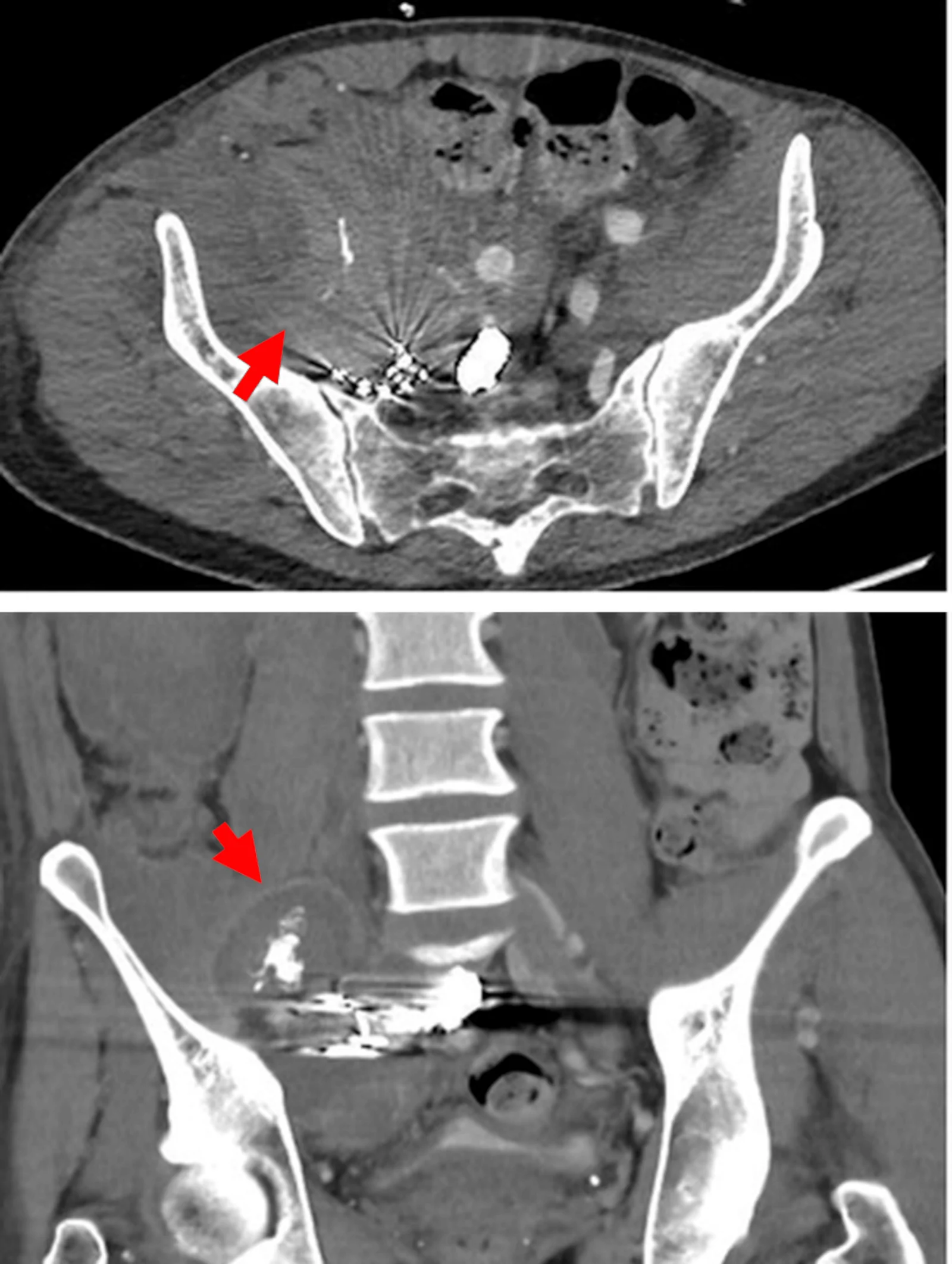
Postoperative computed tomography after endovascular repair CT: computed tomography; NBCA: n-butyl-2-cyanoacrylate Contrast-enhanced CT obtained after combined coil and NBCA embolization, showing no significant enlargement of the retroperitoneal hematoma and no evidence of active contrast extravasation. The red arrow indicates the ruptured internal iliac artery aneurysm occluded by coil embolization and NBCA

The patient's hemodynamic status stabilized postoperatively. Follow-up CT showed no enlargement of the retroperitoneal hematoma or contrast extravasation, and the aneurysm was thrombosed (Figure 5). Although sensory dysesthesia and mild paresis of the right lower extremity persisted, these improved with rehabilitation, and the patient was discharged ambulatory on postprocedure day 30. Subsequently, based on his physical findings and family history, genetic testing was performed and revealed a TGFBR1 gene mutation, confirming the diagnosis of LDS.

## Discussion

LDS is an autosomal dominant hereditary connective tissue disorder caused by mutations in genes related to TGF-β signaling, characterized by systemic arterial aneurysm formation, vessel tortuosity, and dissection [1-3]. In particular, cases with TGFBR1/2 mutations are known to present with early-onset, rapidly progressive vascular lesions, and, importantly, rupture and/or dissection can occur in smaller vessels than in Marfan syndrome [1]. Our patient, at 38 years of age, presented with multiple systemic aneurysms, diffuse arterial irregularity, features characteristic of LDS (hypertelorism, arachnodactyly, and joint hypermobility), and a positive family history, consistent with a typical LDS phenotype. Subsequent genetic testing identified a missense mutation in the TGFBR1 gene, c.605C>T (p.Ala202Val). This variant has been associated with LDS type 1 (LDS1) and is thought to contribute to vascular pathology through impaired TGF-β receptor function [2].

While aortic involvement in LDS has received considerable attention, peripheral arterial lesions are also frequently observed [3,4]. In our patient, aneurysms were identified across an extensive vascular distribution, including the aortic root, abdominal aorta, internal thoracic arteries, hepatic artery, superior mesenteric artery, and superior and inferior gluteal arteries. This distribution reflects the systemic arterial fragility seen in LDS. To the extent of our literature search, reports of internal iliac artery aneurysm as an LDS-related lesion are relatively rare. Although surgical treatment has been described in such cases [5,6], no reports of rupture were identified.

Regarding treatment of aneurysms in LDS, case reports describe thoracic or abdominal aortic stent-graft placement [7-9]; however, the indication for endovascular treatment in LDS remains a subject of debate [10]. Given the marked vascular wall fragility in LDS, concerns exist that the chronic radial force exerted by stent-grafts may cause landing zone expansion, new aneurysmal formation, endoleak, or new dissection [3]. Catheter and guidewire manipulation are also considered to carry a higher risk for vascular injury, perforation, and iatrogenic dissection than in the general population. For these reasons, open surgical reconstruction, such as graft replacement, is often favored as a first-line treatment in younger patients from the standpoint of durability [1].

On the other hand, conventional surgical reconstruction or hemostatic procedures for internal iliac artery aneurysms are often anatomically challenging. Recent European Society for Vascular Surgery (ESVS) guidelines indicate that endovascular treatment may be considered first-line when anatomically feasible [11,12]. According to the 2019 ESVS clinical practice guidelines on abdominal aorto-iliac artery aneurysms, elective repair of an isolated iliac artery aneurysm is generally recommended once the diameter reaches 3.5 cm [12]; however, our patient's aneurysm had not been previously identified or placed under surveillance, and presented instead with rupture, underscoring the unpredictable rupture risk associated with peripheral aneurysms in LDS, which are known to rupture at smaller diameters than in the general population [1]. Reported endovascular approaches to ruptured internal iliac artery aneurysms include coil embolization and stent-graft placement [13-15], with favorable outcomes achieved in both approaches through embolization of distal branch vessels combined with proximal inflow occlusion.

NBCA has previously been reported for intra-aneurysmal embolization in ruptured aortic and iliac artery aneurysms [16,17]. NBCA embolization does not require selective catheterization of small distal vessels, since the agent is carried by blood flow to the target vessel, and its embolic effect does not depend on coagulation status. This makes it a reliable option for rapid hemostasis even in patients with coagulopathy or hemorrhagic shock [16]. However, because the extent of NBCA distribution is influenced by hemodynamics, viscosity (concentration), and injection rate, careful technique is required to avoid unintended embolization of non-targeted vessels.

In our case, given that an adequate neck was present in the internal iliac artery, coil embolization was judged anatomically suitable for hemostasis. Because the distal branch vessel (superior gluteal artery) was involved in the aneurysm measuring 88 mm, including the rupture site, rapid coil embolization via catheter was considered technically difficult. As the superior gluteal artery was the only distal branch involved, we judged that even unintended embolization would have limited impact on the overall prognosis, and therefore prioritized hemostasis by selecting NBCA embolization. A stent-graft was kept on standby in case the proximal neck coil embolization failed to achieve adequate flow occlusion. Although the diagnosis of LDS was confirmed only after the emergency intervention, the patient's phenotypic features (hypertelorism, arachnodactyly, and joint hypermobility), family history of vascular death, and the diffuse multiplicity of aneurysms noted on preoperative imaging raised suspicion of an underlying connective tissue disorder, and catheter manipulation was performed with corresponding caution. A higher NBCA-to-Lipiodol ratio was used to limit the extent of embolization. Intraoperative angiography confirmed embolization of both the proximal and distal regions of the rupture site with satisfactory hemostasis, and no unintended embolization to other vascular territories occurred. Postoperative CT showed no contrast enhancement at the rupture site, and the patient's condition remained stable. The postoperative course was otherwise uneventful aside from a degree of neurological deficit of the right lower extremity due to retroperitoneal hematoma compression, which required rehabilitation. In cases of a ruptured internal iliac artery aneurysm with hemodynamic collapse, such as ours, rapid hemorrhage control is essential. Careful patient selection and combined NBCA and coil embolization can be an effective option for emergency hemostasis. As clinical and imaging follow-up in this case was limited to 30 days, this conclusion should be restricted to successful emergency hemostasis; longer-term follow-up will be required to establish the durability of this approach, including surveillance for recanalization, coil compaction or migration, and collateral reperfusion of the aneurysm sac. To the best of our knowledge, no prior report has described hemostasis achieved with NBCA and coil embolization for a ruptured internal iliac artery aneurysm, regardless of underlying LDS.

Because this patient has LDS, there is a heightened long-term risk of recanalization of the embolized segment, coil compaction or migration, and collateral reperfusion of the aneurysm sac, as well as progression or new aneurysm formation elsewhere in the arterial tree; careful long-term clinical and imaging follow-up is therefore necessary to detect persistent or recurrent perfusion of the aneurysm sac. Close surveillance and consideration of treatment intervention are also required for the patient's potential other systemic multiple arterial lesions. As the aortic root has already dilated to 48 mm, a diameter that approaches or meets thresholds at which prophylactic surgical repair is generally considered in patients with connective tissue disorders and aortopathy of other etiologies [18], surgical treatment is planned; because the natural history of TGFBR-related vasculopathy is more aggressive than that of the general population, intervention thresholds in LDS are typically lower than those applied in other aortopathies [1]. For the remaining multiple peripheral aneurysms, we plan to continue strict follow-up with beta-blocker and angiotensin-converting enzyme inhibitor/angiotensin receptor blocker (ACEI/ARB) therapy, and in the future to determine the optimal treatment strategy for each lesion based on its location, anatomical characteristics, and the patient's overall condition.

## Conclusions

We report a rare case of young-onset ruptured internal iliac artery aneurysm secondary to LDS, in which the patient was successfully rescued by emergency endovascular treatment using NBCA and coil embolization, achieving successful hemostasis at 30-day follow-up. Continued systemic vascular surveillance, including the treated site, remains essential going forward.
